# Supplementary material for: A novel computational approach to the silencing of Sugarcane Bacilliform Guadeloupe A Virus determines potential host-derived MicroRNAs in sugarcane (Saccharum officinarum L.)
Source: PeerJ. 2020 Jan 13;8:e8359. doi: 10.7717/peerj.8359 (PMC6964690; doi:10.7717/peerj.8359)
Supplement: File S1 [file peerj-08-8359-s001.pdf]

```
============================================================
miranda v3.3a    microRNA Target Scanning Algorithm
============================================================
(c) 2003 Memorial Sloan-Kettering Cancer Center, New York

Authors: Anton Enright, Bino John, Chris Sander and Debora Marks
(mirnatargets (at) cbio.mskcc.org - reaches all authors)

Software written by: Anton Enright
Distributed for anyone to use under the GNU Public License (GPL),
See the files 'COPYING' and 'LICENSE' for details

If you use this software please cite:
Enright AJ, John B, Gaul U, Tuschl T, Sander C and Marks DS;
(2003) Genome Biology; 5(1):R1.

   miranda comes with ABSOLUTELY NO WARRANTY;
   This is free software, and you are welcome to redistribute it
   under certain conditions; type `miranda --license' for details.

Current Settings:
============================================================
Query Filename: ./seq/mirna/sof_mirna.fasta
Reference Filename:   ./seq/gene/sof/NC_038382.fasta
Gap Open Penalty:     -9.000000
Gap Extend Penalty:   -4.000000
Score Threshold: 130.000000
Energy Threshold:     -15.000000 kcal/mol
Scaling Parameter:    4.000000
============================================================
Read Sequence:sof-miR156 (20 nt)
Read Sequence:NC_038382.1 Sugarcane bacilliform Guadeloupe A virus
clone 1, complete genome
(7444 nt)
============================================================
Performing Scan: sof-miR156 vs NC_038382.1
============================================================
Score for this Scan:
No Hits Found above Threshold
Complete

Read Sequence:sof-miR159a (21 nt)
Read Sequence:NC_038382.1 Sugarcane bacilliform Guadeloupe A virus
clone 1, complete genome
(7444 nt)
============================================================
Performing Scan: sof-miR159a vs NC_038382.1
============================================================

   Forward:     Score: 138.000000  Q:2 to 19  R:5282 to 5302 Align Len
(17) (64.71%) (76.47%)
```

```
   Query:    3' gucUCGAGGGAAGUUAGGUUu 5'
                  ||  ||:  |||||:||
   Ref:      5' attAGACCCTCACAATCTAAa 3'

   Energy:  -17.180000 kCal/Mol

Scores for this hit:
>sof-miR159a    NC_038382.1     138.00     -17.18     2 19  5282 5302
     17   64.71%     76.47%


   Forward:      Score: 131.000000  Q:2 to 20  R:6739 to 6759 Align Len
(18) (55.56%) (72.22%)

   Query:    3' guCUCGAGGGAAGUUAGGUUu 5'
                 |: | :||   |||||:|
   Ref:      5' caGGACATCCAGAAATCCGAa 3'

   Energy:  -18.110001 kCal/Mol

Scores for this hit:
>sof-miR159a    NC_038382.1     131.00     -18.11     2 20  6739 6759
     18   55.56%     72.22%

Score for this Scan:
Seq1,Seq2,Tot Score,Tot Energy,Max Score,Max
Energy,Strand,Len1,Len2,Positions
>>sof-miR159a   NC_038382.1     269.00     -35.29     138.00     -
18.11 2    21   7444   5282 6739
Complete

Read Sequence:sof-miR159b (21 nt)
Read Sequence:NC_038382.1 Sugarcane bacilliform Guadeloupe A virus
clone 1, complete genome
(7444 nt)
=-=-=-=-=-=-=-=-=-=-=-=-=-=-=-=-=-=-=-=-=-=-=-=-=-=-=-=-=-=-=
Performing Scan: sof-miR159b vs NC_038382.1
=-=-=-=-=-=-=-=-=-=-=-=-=-=-=-=-=-=-=-=-=-=-=-=-=-=-=-=-=-=-=

   Forward:      Score: 138.000000  Q:2 to 19  R:5282 to 5302 Align Len
(17) (64.71%) (76.47%)

   Query:    3' gucUCGAGGGAAGUUAGGUUu 5'
                  ||  ||:  |||||:||
   Ref:      5' attAGACCCTCACAATCTAAa 3'

   Energy:  -17.180000 kCal/Mol

Scores for this hit:
>sof-miR159b    NC_038382.1     138.00     -17.18     2 19  5282 5302
     17   64.71%     76.47%
```

```
   Forward:     Score: 131.000000  Q:2 to 20  R:6739 to 6759 Align Len
(18) (55.56%) (72.22%)

   Query:    3' guCUCGAGGGAAGUUAGGUUu 5'
                 |: | :||   |||||:|
   Ref:      5' caGGACATCCAGAAATCCGAa 3'

   Energy:  -18.110001 kCal/Mol

Scores for this hit:
>sof-miR159b    NC_038382.1     131.00     -18.11     2 20  6739 6759
     18   55.56%     72.22%

Score for this Scan:
Seq1,Seq2,Tot Score,Tot Energy,Max Score,Max
Energy,Strand,Len1,Len2,Positions
>>sof-miR159b   NC_038382.1     269.00     -35.29     138.00     -
18.11 3    21   7444  5282 6739
Complete

Read Sequence:sof-miR159c (21 nt)
Read Sequence:NC_038382.1 Sugarcane bacilliform Guadeloupe A virus
clone 1, complete genome
(7444 nt)
=========================================================
Performing Scan: sof-miR159c vs NC_038382.1
=========================================================

   Forward:     Score: 131.000000  Q:2 to 20  R:6739 to 6759 Align Len
(18) (55.56%) (72.22%)

   Query:    3' ucCUCGAGGGAAGUUAGGUUc 5'
                 |: | :||   |||||:|
   Ref:      5' caGGACATCCAGAAATCCGAa 3'

   Energy:  -16.700001 kCal/Mol

Scores for this hit:
>sof-miR159c    NC_038382.1     131.00     -16.70     2 20  6739 6759
     18   55.56%     72.22%

Score for this Scan:
Seq1,Seq2,Tot Score,Tot Energy,Max Score,Max
Energy,Strand,Len1,Len2,Positions
>>sof-miR159c   NC_038382.1     131.00     -16.70     131.00     -
16.70 4    21   7444  6739
Complete

Read Sequence:sof-miR159d (21 nt)
Read Sequence:NC_038382.1 Sugarcane bacilliform Guadeloupe A virus
clone 1, complete genome
```

```
(7444 nt)
=-=-=-=-=-=-=-=-=-=-=-=-=-=-=-=-=-=-=-=-=-=-=-=-=-=-=-=-=-=-=-
Performing Scan: sof-miR159d vs NC_038382.1
=-=-=-=-=-=-=-=-=-=-=-=-=-=-=-=-=-=-=-=-=-=-=-=-=-=-=-=-=-=-=-

   Forward:     Score: 138.000000  Q:2 to 19  R:5282 to 5302 Align Len
(17) (64.71%) (76.47%)

   Query:    3' gucUCGAGGGAAGUUAGGUUu 5'
                   ||  ||:  |||||:||
   Ref:      5' attAGACCCTCACAATCTAAa 3'

   Energy:  -17.180000 kCal/Mol

Scores for this hit:
>sof-miR159d    NC_038382.1     138.00     -17.18     2 19  5282 5302
     17   64.71%     76.47%


   Forward:     Score: 131.000000  Q:2 to 20  R:6739 to 6759 Align Len
(18) (55.56%) (72.22%)

   Query:    3' guCUCGAGGGAAGUUAGGUUu 5'
                  |: | :||   |||||:|
   Ref:      5' caGGACATCCAGAAATCCGAa 3'

   Energy:  -18.110001 kCal/Mol

Scores for this hit:
>sof-miR159d    NC_038382.1     131.00     -18.11     2 20  6739 6759
     18   55.56%     72.22%

Score for this Scan:
Seq1,Seq2,Tot Score,Tot Energy,Max Score,Max
Energy,Strand,Len1,Len2,Positions
>>sof-miR159d   NC_038382.1     269.00     -35.29     138.00     -
18.11 5    21   7444  5282 6739
Complete

Read Sequence:sof-miR159e (21 nt)
Read Sequence:NC_038382.1 Sugarcane bacilliform Guadeloupe A virus
clone 1, complete genome
(7444 nt)
=-=-=-=-=-=-=-=-=-=-=-=-=-=-=-=-=-=-=-=-=-=-=-=-=-=-=-=-=-=-=-
Performing Scan: sof-miR159e vs NC_038382.1
=-=-=-=-=-=-=-=-=-=-=-=-=-=-=-=-=-=-=-=-=-=-=-=-=-=-=-=-=-=-=-

   Forward:     Score: 142.000000  Q:2 to 19  R:5282 to 5302 Align Len
(17) (70.59%) (76.47%)

   Query:    3' uucUCGAGGAAAGUUAGGUUu 5'
                   ||  |||  |||||:||
```

```
   Ref:      5' attAGACCCTCACAATCTAAa 3'

   Energy:  -15.810000 kCal/Mol

Scores for this hit:
>sof-miR159e    NC_038382.1     142.00     -15.81     2 19  5282 5302
     17   70.59%     76.47%

Score for this Scan:
Seq1,Seq2,Tot Score,Tot Energy,Max Score,Max
Energy,Strand,Len1,Len2,Positions
>>sof-miR159e   NC_038382.1     142.00     -15.81     142.00     -
15.81 6    21   7444  5282
Complete

Read Sequence:sof-miR167a (21 nt)
Read Sequence:NC_038382.1 Sugarcane bacilliform Guadeloupe A virus
clone 1, complete genome
(7444 nt)
=-=-=-=-=-=-=-=-=-=-=-=-=-=-=-=-=-=-=-=-=-=-=-=-=-=-=-=-=-=-=-=-=
Performing Scan: sof-miR167a vs NC_038382.1
=-=-=-=-=-=-=-=-=-=-=-=-=-=-=-=-=-=-=-=-=-=-=-=-=-=-=-=-=-=-=-=-=

   Forward:      Score: 132.000000  Q:2 to 19  R:5846 to 5865 Align Len
(17) (76.47%) (82.35%)

   Query:    3' gucUAGUACGACCGUCGAAGu 5'
                  |||| |:| ||||| ||
   Ref:      5' aaaATCAAGTT-GCAGCCTCa 3'

   Energy:  -16.080000 kCal/Mol

Scores for this hit:
>sof-miR167a    NC_038382.1     132.00     -16.08     2 19  5846 5865
     17   76.47%     82.35%

Score for this Scan:
Seq1,Seq2,Tot Score,Tot Energy,Max Score,Max
Energy,Strand,Len1,Len2,Positions
>>sof-miR167a   NC_038382.1     132.00     -16.08     132.00     -
16.08 7    21   7444  5846
Complete

Read Sequence:sof-miR167b (21 nt)
Read Sequence:NC_038382.1 Sugarcane bacilliform Guadeloupe A virus
clone 1, complete genome
(7444 nt)
=-=-=-=-=-=-=-=-=-=-=-=-=-=-=-=-=-=-=-=-=-=-=-=-=-=-=-=-=-=-=-=-=
Performing Scan: sof-miR167b vs NC_038382.1
=-=-=-=-=-=-=-=-=-=-=-=-=-=-=-=-=-=-=-=-=-=-=-=-=-=-=-=-=-=-=-=-=
```

```
   Forward:     Score: 132.000000  Q:2 to 19  R:5846 to 5865 Align Len
(17) (76.47%) (82.35%)

   Query:    3' gucUAGUACGACCGUCGAAGu 5'
                  |||| |:| ||||| ||
   Ref:      5' aaaATCAAGTT-GCAGCCTCa 3'

   Energy:  -16.080000 kCal/Mol

Scores for this hit:
>sof-miR167b    NC_038382.1     132.00     -16.08     2 19  5846 5865
     17   76.47%     82.35%

Score for this Scan:
Seq1,Seq2,Tot Score,Tot Energy,Max Score,Max
Energy,Strand,Len1,Len2,Positions
>>sof-miR167b   NC_038382.1     132.00     -16.08     132.00     -
16.08 8    21   7444   5846
Complete

Read Sequence:sof-miR168a (21 nt)
Read Sequence:NC_038382.1 Sugarcane bacilliform Guadeloupe A virus
clone 1, complete genome
(7444 nt)
=-=-=-=-=-=-=-=-=-=-=-=-=-=-=-=-=-=-=-=-=-=-=-=-=-=-=-=-=-=
Performing Scan: sof-miR168a vs NC_038382.1
=-=-=-=-=-=-=-=-=-=-=-=-=-=-=-=-=-=-=-=-=-=-=-=-=-=-=-=-=-=

   Forward:     Score: 130.000000  Q:2 to 17  R:5506 to 5525 Align Len
(15) (73.33%) (93.33%)

   Query:    3' cagggCUAGACGUGGUUCGCu 5'
                    |||:| |||||:|:|
   Ref:      5' gagtgGATTT-CACCAGGTGg 3'

   Energy:  -21.240000 kCal/Mol

Scores for this hit:
>sof-miR168a    NC_038382.1     130.00     -21.24     2 17  5506 5525
     15   73.33%     93.33%


   Forward:     Score: 130.000000  Q:2 to 18  R:7050 to 7068 Align Len
(16) (68.75%) (75.00%)

   Query:    3' caggGCUAGACGUGGUUCGCu 5'
                   |||  ||  |||||:|
   Ref:      5' gttaCGAGATG--CCAAGTGa 3'

   Energy:  -18.459999 kCal/Mol

Scores for this hit:
```

```
>sof-miR168a    NC_038382.1     130.00     -18.46     2 18  7050 7068
     16   68.75%     75.00%

Score for this Scan:
Seq1,Seq2,Tot Score,Tot Energy,Max Score,Max
Energy,Strand,Len1,Len2,Positions
>>sof-miR168a   NC_038382.1     260.00     -39.70     130.00     -
21.24 9    21   7444   5506 7050
Complete

Read Sequence:sof-miR168b (20 nt)
Read Sequence:NC_038382.1 Sugarcane bacilliform Guadeloupe A virus
clone 1, complete genome
(7444 nt)
===========================================================
Performing Scan: sof-miR168b vs NC_038382.1
===========================================================

   Forward:     Score: 134.000000  Q:2 to 17  R:7050 to 7068 Align Len
(15) (73.33%) (80.00%)

   Query:    3' caggGCUAGACGGGUUCGCu 5'
                    |||  || |||||:|
   Ref:      5' gttaCGAGATG-CCAAGTGa 3'

   Energy:  -20.059999 kCal/Mol

Scores for this hit:
>sof-miR168b    NC_038382.1     134.00     -20.06     2 17  7050 7068
     15   73.33%     80.00%

Score for this Scan:
Seq1,Seq2,Tot Score,Tot Energy,Max Score,Max
Energy,Strand,Len1,Len2,Positions
>>sof-miR168b   NC_038382.1     134.00     -20.06     134.00     -
20.06 10   20   7444   7050
Complete

Read Sequence:sof-miR396 (21 nt)
Read Sequence:NC_038382.1 Sugarcane bacilliform Guadeloupe A virus
clone 1, complete genome
(7444 nt)
===========================================================
Performing Scan: sof-miR396 vs NC_038382.1
===========================================================

   Forward:     Score: 158.000000  Q:2 to 20  R:3394 to 3415 Align Len
(19) (68.42%) (84.21%)

   Query:    3' guCAAGUUC-UUUCGACACCUu 5'
                  | |:|:| :| ||||||||
   Ref:      5' agGATTAGGTGATGCTGTGGAg 3'
```

Energy: -19.990000 kCal/Mol

Scores for this hit:
>sof-miR396 NC_038382.1 158.00 -19.99 2 20 3394 3415 19 68.42% 84.21%

Score for this Scan:
Seq1,Seq2,Tot Score,Tot Energy,Max Score,Max Energy,Strand,Len1,Len2,Positions
>>sof-miR396 NC_038382.1 158.00 -19.99 158.00 -19.99 11 21 7444 3394
Complete

Read Sequence:sof-miR408a (21 nt)
Read Sequence:NC_038382.1 Sugarcane bacilliform Guadeloupe A virus clone 1, complete genome
(7444 nt)
===
Performing Scan: sof-miR408a vs NC_038382.1
===
Score for this Scan:
No Hits Found above Threshold
Complete

Read Sequence:sof-miR408b (21 nt)
Read Sequence:NC_038382.1 Sugarcane bacilliform Guadeloupe A virus clone 1, complete genome
(7444 nt)
===
Performing Scan: sof-miR408b vs NC_038382.1
===
Score for this Scan:
No Hits Found above Threshold
Complete

Read Sequence:sof-miR408c (21 nt)
Read Sequence:NC_038382.1 Sugarcane bacilliform Guadeloupe A virus clone 1, complete genome
(7444 nt)
===
Performing Scan: sof-miR408c vs NC_038382.1
===
Score for this Scan:
No Hits Found above Threshold
Complete

Read Sequence:sof-miR408d (21 nt)
Read Sequence:NC_038382.1 Sugarcane bacilliform Guadeloupe A virus clone 1, complete genome
(7444 nt)
===

```
Performing Scan: sof-miR408d vs NC_038382.1
===============================================================
Score for this Scan:
No Hits Found above Threshold
Complete

Read Sequence:sof-miR408e (21 nt)
Read Sequence:NC_038382.1 Sugarcane bacilliform Guadeloupe A virus
clone 1, complete genome
(7444 nt)
===============================================================
Performing Scan: sof-miR408e vs NC_038382.1
===============================================================
Score for this Scan:
No Hits Found above Threshold
Complete

Read Sequence:ssp-miR166 (21 nt)
Read Sequence:NC_038382.1 Sugarcane bacilliform Guadeloupe A virus
clone 1, complete genome
(7444 nt)
===============================================================
Performing Scan: ssp-miR166 vs NC_038382.1
===============================================================

   Forward:     Score: 144.000000  Q:2 to 18  R:3249 to 3271 Align Len
(18) (72.22%) (83.33%)

   Query:    3' ccccUUACU--UCGGACCAGGCu 5'
                    | |||  ||:||||||:|
   Ref:      5' agacACTGAAGAGTCTGGTCTGt 3'

   Energy:  -21.450001 kCal/Mol

Scores for this hit:
>ssp-miR166     NC_038382.1     144.00     -21.45     2 18  3249 3271
     18   72.22%     83.33%


   Forward:     Score: 139.000000  Q:2 to 20  R:5589 to 5609 Align Len
(18) (61.11%) (77.78%)

   Query:    3' ccCCUUACUUCGGACCAGGCu 5'
                  | ||||   ::|||||:|
   Ref:      5' taGTAATGCCATTTGGTCTGa 3'

   Energy:  -17.549999 kCal/Mol

Scores for this hit:
>ssp-miR166     NC_038382.1     139.00     -17.55     2 20  5589 5609
     18   61.11%     77.78%
```

```
   Forward:     Score: 132.000000  Q:2 to 14  R:94 to 116 Align Len
(14) (71.43%) (85.71%)

   Query:    3' ccccuuacUUCGG--ACCAGGCu 5'
                        |||:|  ||||:||
   Ref:      5' agctgtacAAGTCTATGGTTCGa 3'

   Energy:  -16.670000 kCal/Mol

Scores for this hit:
>ssp-miR166     NC_038382.1     132.00     -16.67     2 14  94 116
     14   71.43%     85.71%


   Forward:     Score: 132.000000  Q:3 to 20  R:5761 to 5779 Align Len
(17) (76.47%) (88.24%)

   Query:    3' ccCCUUACUUCGGACCAGGcu 5'
                  ||||||  |:||||||:
   Ref:      5' aaGGAATG--GTCTGGTCTta 3'

   Energy:  -21.740000 kCal/Mol

Scores for this hit:
>ssp-miR166     NC_038382.1     132.00     -21.74     3 20  5761 5779
     17   76.47%     88.24%

Score for this Scan:
Seq1,Seq2,Tot Score,Tot Energy,Max Score,Max
Energy,Strand,Len1,Len2,Positions
>>ssp-miR166    NC_038382.1     547.00     -77.41     144.00     -
21.74 17   21   7444  3249 5589 94 5761
Complete

Read Sequence:ssp-miR169 (21 nt)
Read Sequence:NC_038382.1 Sugarcane bacilliform Guadeloupe A virus
clone 1, complete genome
(7444 nt)
=-=-=-=-=-=-=-=-=-=-=-=-=-=-=-=-=-=-=-=-=-=-=-=-=-=-=-=-=-=-=-=-=-=-=
Performing Scan: ssp-miR169 vs NC_038382.1
=-=-=-=-=-=-=-=-=-=-=-=-=-=-=-=-=-=-=-=-=-=-=-=-=-=-=-=-=-=-=-=-=-=-=
Score for this Scan:
No Hits Found above Threshold
Complete

Read Sequence:ssp-miR437a (21 nt)
Read Sequence:NC_038382.1 Sugarcane bacilliform Guadeloupe A virus
clone 1, complete genome
(7444 nt)
=-=-=-=-=-=-=-=-=-=-=-=-=-=-=-=-=-=-=-=-=-=-=-=-=-=-=-=-=-=-=-=-=-=-=
Performing Scan: ssp-miR437a vs NC_038382.1
```

```
============================================================
Score for this Scan:
No Hits Found above Threshold
Complete

Read Sequence:ssp-miR437b (21 nt)
Read Sequence:NC_038382.1 Sugarcane bacilliform Guadeloupe A virus
clone 1, complete genome
(7444 nt)
============================================================
Performing Scan: ssp-miR437b vs NC_038382.1
============================================================
Score for this Scan:
No Hits Found above Threshold
Complete

Read Sequence:ssp-miR437c (21 nt)
Read Sequence:NC_038382.1 Sugarcane bacilliform Guadeloupe A virus
clone 1, complete genome
(7444 nt)
============================================================
Performing Scan: ssp-miR437c vs NC_038382.1
============================================================
Score for this Scan:
No Hits Found above Threshold
Complete

Read Sequence:ssp-miR528 (21 nt)
Read Sequence:NC_038382.1 Sugarcane bacilliform Guadeloupe A virus
clone 1, complete genome
(7444 nt)
============================================================
Performing Scan: ssp-miR528 vs NC_038382.1
============================================================

   Forward:     Score: 147.000000  Q:2 to 18  R:7426 to 7444 Align Len
(16) (75.00%) (75.00%)

   Query:    3' gaggAGACGUACGGGGAAGGu 5'
                    || ||   ||||||||
   Ref:      5' gcgaTCCGC-CCCCCCTTCC- 3'

   Energy:  -18.370001 kCal/Mol

Scores for this hit:
>ssp-miR528     NC_038382.1     147.00     -18.37     2 18  7426 7444
     16   75.00%     75.00%


   Forward:     Score: 133.000000  Q:2 to 15  R:7395 to 7417 Align Len
(15) (73.33%) (80.00%)
```

```
   Query:    3' gaggagaCGUAC--GGGGAAGGu 5'
                       ||| |  ||:|||||
   Ref:      5' gtagagcGCAAGATCCTCTTCCg 3'

   Energy:  -18.059999 kCal/Mol

Scores for this hit:
>ssp-miR528     NC_038382.1     133.00     -18.06     2 15  7395 7417
     15   73.33%     80.00%

Score for this Scan:
Seq1,Seq2,Tot Score,Tot Energy,Max Score,Max
Energy,Strand,Len1,Len2,Positions
>>ssp-miR528    NC_038382.1     280.00     -36.43     147.00     -
18.37 22   21   7444   7426 7395
Complete

Read Sequence:ssp-miR827 (21 nt)
Read Sequence:NC_038382.1 Sugarcane bacilliform Guadeloupe A virus
clone 1, complete genome
(7444 nt)
=-=-=-=-=-=-=-=-=-=-=-=-=-=-=-=-=-=-=-=-=-=-=-=-=-=-=-=-=-=-=-=-=
Performing Scan: ssp-miR827 vs NC_038382.1
=-=-=-=-=-=-=-=-=-=-=-=-=-=-=-=-=-=-=-=-=-=-=-=-=-=-=-=-=-=-=-=-=

   Forward:     Score: 136.000000  Q:2 to 17  R:5378 to 5398 Align Len
(15) (80.00%) (86.67%)

   Query:    3' acaaaCGACUACCAGUAGAUu 5'
                     ||||:|| ||| |||
   Ref:      5' gaaagGCTGGTGTTCAACTAt 3'

   Energy:  -16.400000 kCal/Mol

Scores for this hit:
>ssp-miR827     NC_038382.1     136.00     -16.40     2 17  5378 5398
     15   80.00%     86.67%

Score for this Scan:
Seq1,Seq2,Tot Score,Tot Energy,Max Score,Max
Energy,Strand,Len1,Len2,Positions
>>ssp-miR827    NC_038382.1     136.00     -16.40     136.00     -
16.40 23   21   7444   5378
Complete

Read Sequence:ssp-miR444a (21 nt)
Read Sequence:NC_038382.1 Sugarcane bacilliform Guadeloupe A virus
clone 1, complete genome
(7444 nt)
=-=-=-=-=-=-=-=-=-=-=-=-=-=-=-=-=-=-=-=-=-=-=-=-=-=-=-=-=-=-=-=-=
Performing Scan: ssp-miR444a vs NC_038382.1
=-=-=-=-=-=-=-=-=-=-=-=-=-=-=-=-=-=-=-=-=-=-=-=-=-=-=-=-=-=-=-=-=
```

```
Score for this Scan:
No Hits Found above Threshold
Complete

Read Sequence:ssp-miR444b (21 nt)
Read Sequence:NC_038382.1 Sugarcane bacilliform Guadeloupe A virus
clone 1, complete genome
(7444 nt)
=-=-=-=-=-=-=-=-=-=-=-=-=-=-=-=-=-=-=-=-=-=-=-=-=-=-=-=-=-=-=-=-=
Performing Scan: ssp-miR444b vs NC_038382.1
=-=-=-=-=-=-=-=-=-=-=-=-=-=-=-=-=-=-=-=-=-=-=-=-=-=-=-=-=-=-=-=-=
Score for this Scan:
No Hits Found above Threshold
Complete

Read Sequence:ssp-miR444c-3p (21 nt)
Read Sequence:NC_038382.1 Sugarcane bacilliform Guadeloupe A virus
clone 1, complete genome
(7444 nt)
=-=-=-=-=-=-=-=-=-=-=-=-=-=-=-=-=-=-=-=-=-=-=-=-=-=-=-=-=-=-=-=-=
Performing Scan: ssp-miR444c-3p vs NC_038382.1
=-=-=-=-=-=-=-=-=-=-=-=-=-=-=-=-=-=-=-=-=-=-=-=-=-=-=-=-=-=-=-=-=
Score for this Scan:
No Hits Found above Threshold
Complete

Read Sequence:ssp-miR1128 (21 nt)
Read Sequence:NC_038382.1 Sugarcane bacilliform Guadeloupe A virus
clone 1, complete genome
(7444 nt)
=-=-=-=-=-=-=-=-=-=-=-=-=-=-=-=-=-=-=-=-=-=-=-=-=-=-=-=-=-=-=-=-=
Performing Scan: ssp-miR1128 vs NC_038382.1
=-=-=-=-=-=-=-=-=-=-=-=-=-=-=-=-=-=-=-=-=-=-=-=-=-=-=-=-=-=-=-=-=

   Forward:     Score: 136.000000  Q:2 to 18  R:6141 to 6163 Align Len
(18) (77.78%) (83.33%)

   Query:    3' aaacCCUGCCU--CCCUCAUCAu 5'
                    |||:|||  ||||| |||
   Ref:      5' gtatGGATGGATGGGGAGGAGTc 3'

   Energy:  -22.969999 kCal/Mol

Scores for this hit:
>ssp-miR1128    NC_038382.1     136.00     -22.97     2 18  6141 6163
     18   77.78%     83.33%

Score for this Scan:
Seq1,Seq2,Tot Score,Tot Energy,Max Score,Max
Energy,Strand,Len1,Len2,Positions
>>ssp-miR1128   NC_038382.1     136.00     -22.97     136.00     -
22.97 27   21   7444   6141
```

```
Complete

Read Sequence:ssp-miR1432 (21 nt)
Read Sequence:NC_038382.1 Sugarcane bacilliform Guadeloupe A virus
clone 1, complete genome
(7444 nt)
=-=-=-=-=-=-=-=-=-=-=-=-=-=-=-=-=-=-=-=-=-=-=-=-=-=-=-=-=-=-=-=-=-=
Performing Scan: ssp-miR1432 vs NC_038382.1
=-=-=-=-=-=-=-=-=-=-=-=-=-=-=-=-=-=-=-=-=-=-=-=-=-=-=-=-=-=-=-=-=-=

   Forward:     Score: 152.000000  Q:2 to 18  R:6070 to 6091 Align Len
(17) (76.47%) (76.47%)

   Query:    3' cagcCACAGUAGA-AAGGACUc 5'
                    ||| ||  | |||||||
   Ref:      5' aattGTGGCAAATCTTCCTGAt 3'

   Energy:  -15.480000 kCal/Mol

Scores for this hit:
>ssp-miR1432    NC_038382.1     152.00     -15.48     2 18  6070 6091
     17   76.47%     76.47%

Score for this Scan:
Seq1,Seq2,Tot Score,Tot Energy,Max Score,Max
Energy,Strand,Len1,Len2,Positions
>>ssp-miR1432   NC_038382.1     152.00     -15.48     152.00     -
15.48 28   21   7444  6070
Complete

Scan Complete


RNA22 algorithm results of SCBGAV-
sof-miR167a     NC_038382.1 Sugarcane bacilliform Guadeloupe A virus
clone target from/to=[1310,1329] -15.80     AACCTCGACTGGTAGTTTCC
     TGAAGCTGCCAGCATGATCTG ....(((.((((((((((.  .)))))))))))..)))....
     14   20   0.162000
sof-miR167b     NC_038382.1 Sugarcane bacilliform Guadeloupe A virus
clone target from/to=[1310, 1329] -15.80     AACCTCGACTGGTAGTTTCC
     TGAAGCTGCCAGCATGATCTG ....(((.((((((((((.  .)))))))))))..)))....
     14   20   0.162000
ssp-miR528 NC_038382.1 Sugarcane bacilliform Guadeloupe A virus clone
     target from/to=[1310,1330] -13.70     AACCTCGACTGGTAGTTTCCA
     TGGAAGGGGCATGCAGAGGAG ..((((.....((..(((((( ))))))..)).....))))..
     12   21   0.162000
sof-miR168a     NC_038382.1 Sugarcane bacilliform Guadeloupe A virus
clone target from/to=[1723,1743] -12.60     AGCAAGAACAGTACCAGGAGA
     TCGCTTGGTGCAGATCGGGAC ..(..((.(.((((((((.(( )).)))))))).).))..)..
     14   21   0.037600
sof-miR408a     NC_038382.1 Sugarcane bacilliform Guadeloupe A virus
clone target from/to=[1735,1753] -13.70     ACCAGGAGAAGTACTGTAT
```

CTGCACTGCCTCTTCCCTGGC .((((((((.(((.((((.   .)))).))).))).)))..))))).
15   19   0.037600
sof-miR408b   NC_038382.1 Sugarcane bacilliform Guadeloupe A virus clone target from/to=[1735,1753] -13.70   ACCAGGAGAAGTACTGTAT
CTGCACTGCCTCTTCCCTGGC .((((((((.(((.((((.   .)))).))).))).)))..))))).
15   19   0.037600
sof-miR408c   NC_038382.1 Sugarcane bacilliform Guadeloupe A virus clone target from/to=[1735,1753] -13.70   ACCAGGAGAAGTACTGTAT
CTGCACTGCCTCTTCCCTGGC .((((((((.(((.((((.   .)))).))).))).)))..))))).
15   19   0.037600
sof-miR408d   NC_038382.1 Sugarcane bacilliform Guadeloupe A virus clone target from/to=[1735,1753] -13.70   ACCAGGAGAAGTACTGTAT
CTGCACTGCCTCTTCCCTGGC .((((((((.(((.((((.   .)))).))).))).)))..))))).
15   19   0.037600
ssp-miR528 NC_038382.1 Sugarcane bacilliform Guadeloupe A virus clone target from/to=[2839,2858] -12.10   AACCACTGAATTCCCATTCA
TGGAAGGGGCATGCAGAGGAG ..((.(((...((((.(((( )))).))))....))).))..
13   20   0.061000
sof-miR396   NC_038382.1 Sugarcane bacilliform Guadeloupe A virus clone target from/to=[2867,2886] -12.70   AGAAGACCGAGAGTGTGGGA
TTCCACAGCTTTCTTGAACTG ....(((((((((((( )))))))).))))).........
12   20   0.102000
sof-miR396   NC_038382.1 Sugarcane bacilliform Guadeloupe A virus clone target from/to=[3394,3415] -17.80   AGGATTAGGTGATGCTGTGGAG
TTCCACAGCTTTCTTGAACTG .((.(((((....(((((((((
)))))))))...))))).)). 16   22   0.030200
sof-miR396   NC_038382.1 Sugarcane bacilliform Guadeloupe A virus clone target from/to=[4115,4133] -13.90   GACTGCAAGAAATGTGGAA
TTCCACAGCTTTCTTGAACTG .....((((((((((((((   )))))))..))))))).....
14   19   0.077600
ssp-miR444a   NC_038382.1 Sugarcane bacilliform Guadeloupe A virus clone target from/to=[4103,4121] -16.30   AAGCACAGGGTTGACTGCA
TGCAGTTGTTGCCTCAAGCTT ((((...((((.(((((((   )))))))...))))...))))
15   19   0.077600
ssp-miR444b   NC_038382.1 Sugarcane bacilliform Guadeloupe A virus clone target from/to=[4103,4121] -16.30   AAGCACAGGGTTGACTGCA
TGCAGTTGTTGCCTCAAGCTT ((((...((((.(((((((   )))))))...))))...))))
15   19   0.077600
ssp-miR444c-3p   NC_038382.1 Sugarcane bacilliform Guadeloupe A virus clone target from/to=[4656,4675] -12.80   CAATCCTAGACACAGGTGCA
TGCAGTTGTTGTCTCAAGCTT .......((((((((.(((( )))).))).))))).......
12   20   0.261000
sof-miR159a   NC_038382.1 Sugarcane bacilliform Guadeloupe A virus clone target from/to=[5067,5085] -12.30   AAGAGTTTATATCTCCAAA
TTTGGATTGAAGGGAGCTCTG .(((((((...((((((((   ))))))..))...))))))).
15   19   0.277000
sof-miR159b   NC_038382.1 Sugarcane bacilliform Guadeloupe A virus clone target from/to=[5067,5085] -12.30   AAGAGTTTATATCTCCAAA
TTTGGATTGAAGGGAGCTCTG .(((((((...((((((((   ))))))..))...))))))).
15   19   0.277000
sof-miR159d   NC_038382.1 Sugarcane bacilliform Guadeloupe A virus clone target from/to=[5067,5085] -12.30   AAGAGTTTATATCTCCAAA

```
     TTTGGATTGAAGGGAGCTCTG .(((((((...((((((((   ))))))..))...))))))).
     15   19   0.277000
sof-miR159e     NC_038382.1 Sugarcane bacilliform Guadeloupe A virus
clone target from/to=[5067,5085] -13.20     AAGAGTTTATATCTCCAAA
     TTTGGATTGAAAGGAGCTCTT ((((((((...((((((((   ))))))..))...))))))))
     16   19   0.277000
sof-miR168a     NC_038382.1 Sugarcane bacilliform Guadeloupe A virus
clone target from/to=[5137,5159] -14.30     GCACTGGGAGAAAAACCAAGTGG
     TCGCTTGGTGCAGATCGGGAC (..((((.......(((((((((
     ))))))))).....))))..) 14   23   0.129000
sof-miR159e     NC_038382.1 Sugarcane bacilliform Guadeloupe A virus
clone target from/to=[5284,5302] -12.40     TAGACCCTCACAATCTAAA
     TTTGGATTGAAAGGAGCTCTT .(((((((..(((((((((   )))))))))..))).).))).
     16   19   0.007820
sof-miR168a     NC_038382.1 Sugarcane bacilliform Guadeloupe A virus
clone target from/to=[5506,5525] -17.70     GAGTGGATTTCACCAGGTGG
     TCGCTTGGTGCAGATCGGGAC ...(.(((((((((((((((  )))))))))).))))).)...
     16   20   0.311000
sof-miR168b     NC_038382.1 Sugarcane bacilliform Guadeloupe A virus
clone target from/to=[5506,5525] -13.70     GAGTGGATTTCACCAGGTGG
     TCGCTTGGGCAGATCGGGAC  ...(.(((((..((((((((  ))))))))..))))).)...
     14   20   0.311000
ssp-miR169      NC_038382.1 Sugarcane bacilliform Guadeloupe A virus
clone target from/to=[6389,6409] -13.30     CCTTCAAGGGTTAGATGGCTA
     TAGCCAAGGATGACTTGCCGG ((..((((.(((...(((((( ))))))..)))..))))..))
     15   21   0.149000
sof-miR408e     NC_038382.1 Sugarcane bacilliform Guadeloupe A virus
clone target from/to=[6985,7003] -12.60     AGCAGTGACGTCAATGCGG
     CTGCACTGACTCTTCCCTGGC ..(((.((.((((.(((((   ))))).))))...)).)))..
     14   19   0.056700
sof-miR168a     NC_038382.1 Sugarcane bacilliform Guadeloupe A virus
clone target from/to=[7012,7030] -12.30     GACCCGAACCACTAGATGG
     TCGCTTGGTGCAGATCGGGAC ..(((((..((((((.(((   ))).))))))....)))))..
     14   19   0.186000
sof-miR168a     NC_038382.1 Sugarcane bacilliform Guadeloupe A virus
clone target from/to=[7050,7068] -17.10     GTTACGAGATGCCAAGTGA
     TCGCTTGGTGCAGATCGGGAC ((..(((..((((((((((   )))))))..)))..)))..))
     15   19   0.347000
sof-miR168b     NC_038382.1 Sugarcane bacilliform Guadeloupe A virus
clone target from/to=[7050,7068] -19.40     GTTACGAGATGCCAAGTGA
     TCGCTTGGGCAGATCGGGAC  ((..(((..((((((((((   )))))).))))..)))..))
     15   19   0.347000
```

Sugarcane bacilliform Guadeloupe A virus
NCBI Reference Sequence: NC_038382.1

dataset: 1
target: NC_038382.1
length: 7444
miRNA : sof-miR156
length: 20
mfe: -23.2 kcal/mol

```
p-value: undefined
position  7104
target 5' C   GUG UG     UAGCUUUAUUCC      U 3'
           UGC   C  UCUCU            UUUGUC
           ACG   G  AGAGA            AGACAG
miRNA  3' C   A   UG                       U 5'
------------------------------------------------------------
dataset: 1
target: NC_038382.1
length: 7444
miRNA : sof-miR159a
length: 21
mfe: -25.4 kcal/mol
p-value: undefined
position 1659
target 5' G     ACA    A   AUGGUUGUAG       C 3'
           GGAGC   CCCU UCA          AUCCAAA
           UCUCG   GGGA AGU          UAGGUUU
miRNA  3' G     A                            5'
------------------------------------------------------------
dataset: 1
target: NC_038382.1
length: 7444
miRNA : sof-miR159b
length: 21
mfe: -25.4 kcal/mol
p-value: undefined
position 1659
target 5' G     ACA    A   AUGGUUGUAG       C 3'
           GGAGC   CCCU UCA          AUCCAAA
           UCUCG   GGGA AGU          UAGGUUU
miRNA  3' G     A                            5'


dataset: 1
target: NC_038382.1
length: 7444
miRNA : sof-miR159c
length: 21
mfe: -28.0 kcal/mol
p-value: undefined

position  6896
target 5' C      CGG  AUA        C 3'
           AGGAGC   CC    GAUCCAGG
           UCCUCG   GG    UUAGGUUC
miRNA  3'        A    GAAG         5'
------------------------------------------------------------
dataset: 1
target: NC_038382.1
length: 7444
miRNA : sof-miR159d
```

```
length: 21
mfe: -25.4 kcal/mol
p-value: undefined
position  1659
target 5' G     ACA    A   AUGGUUGUAG       C 3'
           GGAGC   CCCU UCA          AUCCAAA
           UCUCG   GGGA AGU          UAGGUUU
miRNA  3' G     A                           5'
------------------------------------------------------------
dataset: 1
target: NC_038382.1
length: 7444
miRNA : sof-miR159e
length: 21
mfe: -25.5 kcal/mol
p-value: undefined
position  733
target 5' C    U        GCA       C 3'
           GAGA CUCCUUUU   GAUCCGG
           UUCU GAGGAAAG   UUAGGUU
miRNA  3'      C                  U 5'
------------------------------------------------------------
dataset: 1
target: NC_038382.1
length: 7444
miRNA : sof-miR167a
length: 21
mfe: -26.6 kcal/mol
p-value: undefined
position  1304
target 5' G      ACCUC A           C 3'
           GGGUCA     G CUGGUAGUUUC
           UCUAGU     C GACCGUCGAAG
miRNA  3' G      A                 U 5'

dataset: 1
target: NC_038382.1
length: 7444
miRNA : sof-miR167b
length: 21

mfe: -26.6 kcal/mol
p-value: undefined
position  1304
target 5' G      ACCUC A           C 3'
           GGGUCA     G CUGGUAGUUUC
           UCUAGU     C GACCGUCGAAG
miRNA  3' G      A                 U 5'

dataset: 1
target: NC_038382.1
length: 7444
```

```
miRNA : sof-miR168a
length: 21
mfe: -25.6 kcal/mol
p-value: undefined

position  6084
target 5' U         AGA  U   UCCUGAGA    A  3'
           UCCUGAUCU   GC ACC        AAGC
           AGGGCUAGA   CG UGG        UUCG
miRNA  3' C                              CU 5'


dataset: 1
target: NC_038382.1
length: 7444
miRNA : sof-miR168b
length: 20
mfe: -25.6 kcal/mol
p-value: undefined
position  6937
target 5'  A UU        UGACG     A  3'
            U  GAUCUGCU     UAAGC
            G  CUAGACGG     GUUCG
miRNA  3' CA GG                CU 5'
------------------------------------------------------------
dataset: 1
target: NC_038382.1
length: 7444
miRNA : sof-miR396
length: 21
mfe: -24.9 kcal/mol
p-value: undefined

position  3394
target 5' A  A     UGAU         A 3'
           GG UUAGG    GCUGUGGAG
           UC AGUUC    CGACACCUU
miRNA  3' G  A     UUU            5'
```

psRNATargt-SCBGAV-
miRNA_Acc. Target_Acc. Expectation UPE$ miRNA_start miRNA_end Target_start Target_end miRNA_aligned_fragment Target_aligned_fragment Inhibition Target_Desc. Multiplicity
sof-miR396 NC_038382.1 5.5 -1.0 1 21 5732 5752 UUCCACAGCUUUCUUGAACUG CAUUUGAAGAAAUUUAUGGAA Cleavage Sugarcane bacilliform Guadeloupe A virus 2
sof-miR159a NC_038382.1 6.0 -1.0 1 21 3779 3799 UUUGGAUUGAAGGGAGCUCUG CUGGACUCUCUUGAAUUAGAA Cleavage Sugarcane bacilliform Guadeloupe A virus 2

sof-miR159b NC_038382.1 6.0 -1.0 1 21 3779 3799
UUUGGAUUGAAGGGAGCUCUG CUGGACUCUCUUGAAUUAGAA Cleavage Sugarcane bacilliform Guadeloupe A virus 2
sof-miR159d NC_038382.1 6.0 -1.0 1 21 3779 3799
UUUGGAUUGAAGGGAGCUCUG CUGGACUCUCUUGAAUUAGAA Cleavage Sugarcane bacilliform Guadeloupe A virus 2
sof-miR156 NC_038382.1 6.5 -1.0 1 20 3309 3328
UGACAGAAGAGAGUGAGCAC AUGAUUACUUCCAUUUGUCC Translation
Sugarcane bacilliform Guadeloupe A virus 1
sof-miR159e NC_038382.1 6.5 -1.0 1 21 5282 5302
UUUGGAUUGAAAGGAGCUCUU AUUAGACCCUCACAAUCUAAA Translation
Sugarcane bacilliform Guadeloupe A virus 3
sof-miR159a NC_038382.1 7.0 -1.0 1 21 5282 5302
UUUGGAUUGAAGGGAGCUCUG AUUAGACCCUCACAAUCUAAA Translation
Sugarcane bacilliform Guadeloupe A virus 2
sof-miR159b NC_038382.1 7.0 -1.0 1 21 5282 5302
UUUGGAUUGAAGGGAGCUCUG AUUAGACCCUCACAAUCUAAA Translation
Sugarcane bacilliform Guadeloupe A virus 2
sof-miR159c NC_038382.1 7.0 -1.0 1 21 3779 3799
CUUGGAUUGAAGGGAGCUCCU CUGGACUCUCUUGAAUUAGAA Cleavage Sugarcane bacilliform Guadeloupe A virus 1
sof-miR159d NC_038382.1 7.0 -1.0 1 21 5282 5302
UUUGGAUUGAAGGGAGCUCUG AUUAGACCCUCACAAUCUAAA Translation
Sugarcane bacilliform Guadeloupe A virus 2
sof-miR159e NC_038382.1 7.0 -1.0 1 21 6318 6338
UUUGGAUUGAAAGGAGCUCUU AGAAGGAACUUGUGAUCCGAA Translation
Sugarcane bacilliform Guadeloupe A virus 3
sof-miR167a NC_038382.1 7.0 -1.0 1 21 5846 5865
UGAAGCUGCCAGCAUGAUCUG AAAAUCAAGUUG-CAGCCUCA Cleavage Sugarcane bacilliform Guadeloupe A virus 1
sof-miR167b NC_038382.1 7.0 -1.0 1 21 5846 5865
UGAAGCUGCCAGCAUGAUCUG AAAAUCAAGUUG-CAGCCUCA Cleavage Sugarcane bacilliform Guadeloupe A virus 1
sof-miR159e NC_038382.1 7.5 -1.0 1 21 5590 5610
UUUGGAUUGAAAGGAGCUCUU AGUAAUGCCAUUUGGUCUGAA Cleavage Sugarcane bacilliform Guadeloupe A virus 3
sof-miR396 NC_038382.1 7.5 -1.0 1 21 3394 3415 UUCCACAGC-UUUCUUGAACUG AGGAUUAGGUGAUGCUGUGGAG Cleavage Sugarcane bacilliform Guadeloupe A virus 2
ssp-miR396 NC_038382.1 5.5 -1.0 1 21 5732 5752
UUCCACAGCUUUCUUGAACUG CAUUUGAAGAAAUUUAUGGAA Cleavage Sugarcane bacilliform Guadeloupe A virus 2
ssp-miR444a NC_038382.1 5.5 -1.0 1 21 80 100
UGCAGUUGUUGCCUCAAGCUU UGAUUUGGGUUAAUAGCUGUA Cleavage Sugarcane bacilliform Guadeloupe A virus 3
ssp-miR444b.2 NC_038382.1 5.5 -1.0 1 21 80 100
UGCAGUUGUUGCCUCAAGCUU UGAUUUGGGUUAAUAGCUGUA Cleavage Sugarcane bacilliform Guadeloupe A virus 3
ssp-miR444c-3p NC_038382.1 5.5 -1.0 1 21 80 100
UGCAGUUGUUGUCUCAAGCUU UGAUUUGGGUUAAUAGCUGUA Cleavage Sugarcane bacilliform Guadeloupe A virus 3

ssp-miR159a NC_038382.1 6.0 -1.0 1 21 3779 3799
UUUGGAUUGAAGGGAGCUCUG CUGGACUCUCUUGAAUUAGAA Cleavage Sugarcane
bacilliform Guadeloupe A virus 2
ssp-miR444b.1 NC_038382.1 6.0 -1.0 1 21 756 776
UUGUGGCUUUCUUGCAAGUUG GCAAGAGCAAGAAAGUCAGAA Cleavage Sugarcane
bacilliform Guadeloupe A virus 13
ssp-miR156 NC_038382.1 6.5 -1.0 1 21 3308 3328
UGACAGAAGAGAGUGAGCACA AAUGAUUACUUCCAUUUGUCC Translation
Sugarcane bacilliform Guadeloupe A virus 1
ssp-miR166 NC_038382.1 6.5 -1.0 1 21 981 1001
UCGGACCAGGCUUCAUUCCCC GCAGACUGAGGCUUUGACUGA Cleavage Sugarcane
bacilliform Guadeloupe A virus 1
ssp-miR444b.1 NC_038382.1 6.5 -1.0 1 21 2615 2634
UUGUGGCUUUCUUGCAAGUUG CAAUUU-UUGGAAAGCCUCAA Cleavage Sugarcane
bacilliform Guadeloupe A virus 13
ssp-miR444b.1 NC_038382.1 6.5 -1.0 1 21 5009 5028
UUGUGGCUUUCUUGCAAGUUG GAACU-GCAAGAGUACUAUAA Cleavage Sugarcane
bacilliform Guadeloupe A virus 13
ssp-miR444b.1 NC_038382.1 6.5 -1.0 1 21 5731 5750
UUGUGGCUUUCUUGCAAGUUG GCAUUUG-AAGAAAUUUAUGG Cleavage Sugarcane
bacilliform Guadeloupe A virus 13
ssp-miR444b.1 NC_038382.1 6.5 -1.0 1 21 4471 4490
UUGUGGCUUUCUUGCAAGUUG GUAUUUG-AACAAAAUCACAG Translation
Sugarcane bacilliform Guadeloupe A virus 13
ssp-miR444c-3p NC_038382.1 6.5 -1.0 1 21 4940 4959
UGCAGUUGUUGUCUCAAGCUU AAGAUUAUGACAACAG-UGCA Cleavage Sugarcane
bacilliform Guadeloupe A virus 3
ssp-miR528 NC_038382.1 6.5 -1.0 1 21 2897 2917
UGGAAGGGGCAUGCAGAGGAG GAGUUCUACAUGUUACCUCCA Cleavage Sugarcane
bacilliform Guadeloupe A virus 1
ssp-miR444a NC_038382.1 6.75 -1.0 1 21 775 797
UGCAGUUGUUGC--CUCAAGCUU AAGCGUGAGCUGCAAGAAUUGCA Cleavage
Sugarcane bacilliform Guadeloupe A virus 3
ssp-miR444b.2 NC_038382.1 6.75 -1.0 1 21 775 797
UGCAGUUGUUGC--CUCAAGCUU AAGCGUGAGCUGCAAGAAUUGCA Cleavage
Sugarcane bacilliform Guadeloupe A virus 3
ssp-miR159a NC_038382.1 7.0 -1.0 1 21 5282 5302
UUUGGAUUGAAGGGAGCUCUG AUUAGACCCUCACAAUCUAAA Translation
Sugarcane bacilliform Guadeloupe A virus 2
ssp-miR167b NC_038382.1 7.0 -1.0 1 21 5846 5865
UGAAGCUGCCAGCAUGAUCUG AAAAUCAAGUUG-CAGCCUCA Cleavage Sugarcane
bacilliform Guadeloupe A virus 1
ssp-miR444b.1 NC_038382.1 7.0 -1.0 1 21 5249 5269
UUGUGGCUUUCUUGCAAGUUG AAACAUGUGAAGAAGCUGUUA Translation
Sugarcane bacilliform Guadeloupe A virus 13
ssp-miR444c-3p NC_038382.1 7.25 -1.0 1 21 775 797
UGCAGUUGUUGU--CUCAAGCUU AAGCGUGAGCUGCAAGAAUUGCA Cleavage
Sugarcane bacilliform Guadeloupe A virus 3
ssp-miR396 NC_038382.1 7.5 -1.0 1 21 3394 3415 UUCCACAGC-
UUUCUUGAACUG AGGAUUAGGUGAUGCUGUGGAG Cleavage Sugarcane
bacilliform Guadeloupe A virus 2

ssp-miR444a NC_038382.1 7.5 -1.0 1 21 1463 1482
UGCAGUUGUUGCCUCAAGCUU GAACUUGAAGUAAUGGC-GCA Cleavage Sugarcane bacilliform Guadeloupe A virus 3
ssp-miR444b.1 NC_038382.1 7.5 -1.0 1 21 7034 7056
UUGUGGCUUUCUUGCA--AGUUG UAACUAGUGUGACAGGGUUACGA
Translation Sugarcane bacilliform Guadeloupe A virus 13
ssp-miR444b.1 NC_038382.1 7.5 -1.0 1 21 3494 3515
UUGUGGCUUUCUUGCA-AGUUG GAAUUAUGUGAGAAGGUAGCAU Cleavage
Sugarcane bacilliform Guadeloupe A virus 13
ssp-miR444b.1 NC_038382.1 7.5 -1.0 1 21 3968 3988
UUGUGGCUUUCUUGCAAGUUG UAUUUGGUAGGAGAACCAUCA Cleavage Sugarcane bacilliform Guadeloupe A virus 13
ssp-miR444b.1 NC_038382.1 7.5 -1.0 1 21 7278 7298
UUGUGGCUUUCUUGCAAGUUG CUCUAUAUAAGGAGGCCAUGU Cleavage Sugarcane bacilliform Guadeloupe A virus 13
ssp-miR444b.1 NC_038382.1 7.5 -1.0 1 21 4045 4065
UUGUGGCUUUCUUGCAAGUUG GAGUUUUGAAGAAGCUCAUGU Cleavage Sugarcane bacilliform Guadeloupe A virus 13
ssp-miR444b.1 NC_038382.1 7.5 -1.0 1 21 6099 6119
UUGUGGCUUUCUUGCAAGUUG UACCUCCUGAGAAAGCAAUCA Cleavage Sugarcane bacilliform Guadeloupe A virus 13
ssp-miR444b.1 NC_038382.1 7.5 -1.0 1 21 1193 1212
UUGUGGCUUUCUUGCAAGUUG GCAAUUG-AAUACAGCCAUAU Translation
Sugarcane bacilliform Guadeloupe A virus 13
ssp-miR444b.2 NC_038382.1 7.5 -1.0 1 21 1463 1482
UGCAGUUGUUGCCUCAAGCUU GAACUUGAAGUAAUGGC-GCA Cleavage Sugarcane bacilliform Guadeloupe A virus 3
ssp-miR827 NC_038382.1 7.5 -1.0 1 21 5378 5398
UUAGAUGACCAUCAGCAAACA GAAAGGCUGGUGUUCAACUAU Cleavage Sugarcane bacilliform Guadeloupe A virus 1
